# Comment on “The Use of BRET to Study Receptor-Protein Interactions”

**DOI:** 10.3389/fendo.2014.00003

**Published:** 2014-01-22

**Authors:** Ralf Jockers

**Affiliations:** ^1^U1016, INSERM, Institut Cochin, Paris, France; ^2^CNRS UMR 8104, Paris, France; ^3^University of Paris Descartes, Paris, France

**Keywords:** BRET, high throughput screening assays, GPCR heteromers, PPI inhibitors, protein–protein interactions

This Research Topic assembles for the first time a comprehensive selection of articles (mini review, review articles, original research, and opinion articles) on the bioluminescence resonance energy transfer (BRET) technology. BRET is a natural phenomenon known in several marine organisms. It relies on the non-radiative transfer of energy from an appropriate energy donor to an energy acceptor, provided that both are located at a distance lower than 10 nm [see Ref. (1) for a basic and theoretical introduction]. BRET has been first applied to the detection of protein–protein interactions (PPIs) in 1999 (2). Since then, the technology constantly evolved by combining new donor and acceptor couples and developing various BRET assay formats as discussed in the Mini Review article of De et al. (3).

Many BRET assays have been developed to study the oligomerization of seven-transmembrane-spanning G protein-coupled receptors (GPCRs). The BRET technology provides an attractive way to study this phenomenon in intact cells without the need of solubilization of receptors from their natural membrane environment. The overwhelming majority of BRET-based studies conclude that GPCRs do indeed exist as dimers or higher-order oligomers when transfected into cells at physiological levels. Three of the articles of this Research Topic discuss BRET assays that have been developed to properly address the specificity of BRET signals obtained upon expression of different GPCRs (1, 4, 5). The current consensus confirms that several different BRET assays are needed to evaluate the specificity of BRET signals. The precise role of each of these assays remains a source of controversy in the field (4). Further refinement of BRET control experiments and application of new techniques like single-molecule measurements and functional *in vivo* studies are likely to provide new insights to the existence and physiological relevance of GPCR oligomerization. Not surprisingly, GPCR oligomerization is the main issue of four articles of this Research Topic (4–7) ranging from studies on class A and B GPCR homo- and heteromers using BRET or alternative approaches like time-resolved FRET measurements.

Apart from performing the proper control experiments, another important issue in the BRET field concerns the proper interpretation of stimulus-induced BRET signals. In the context of GPCR oligomerization, agonist-induced BRET signals can be generated by an agonist-driven oligomerization or agonist-induced conformational changes within preassembled oligomers. Discrimination between these two possibilities is not trivial but has become possible due to the development of BRET donor saturation experiments in the absence and presence of receptor stimulation (8, 9). This issue, to which BRET has made a significant contribution, is obviously of general importance for the field of PPIs. This is illustrated by the articles describing the interaction of the protease-activated receptor 1 and 2 with its cognate G proteins as described by Ayoub and Pin (10) and Ayoub et al. (11).

The BRET technology has been extended toward other receptor families like tyrosine kinase receptors and cytokine receptors. This diversification demonstrates the general feature of this technique. These studies did not only address the question of receptor oligomerization but also monitored the real-time interaction of receptors with various effector molecules such as Grb2, PTP1-B, PLC-γ1, etc. This important aspect is discussed in the review article of Siddiqui et al. (12). Receptor–effector interactions have been also monitored by BRET for two privileged GPCR interacting partners, heterotrimeric G proteins and β-arrestins, as documented in articles of this Research Topic (6, 10, 11, 13).

A more recent application of BRET concerns the development of biosensors to monitor downstream events of cellular signaling like the generation of second messengers and activation of intracellular kinases. These sensors are typically composed of the energy donor and acceptor separated by an assay-specific domain that changes its conformation upon second messenger binding or phosphorylation, thus modifying the position of the donor and acceptor and consequently the BRET signal. Similar sensors have been developed for FRET applications, which served as source of inspiration for the development of the BRET sensors. The articles from Salahpour et al. (13) and Xu et al. (2) describe the design and validation of cAMP and ERK sensors.

The high reproducibility of BRET and the robustness of the measurements make BRET an interesting option for the design of high throughput screening assays. Two applications are discussed in this Research Topic. The first concerns the design of an assay for the identification of compounds that specifically activate GPCR heteromers (6) based on the recruitment of β-arrestin to GPCR heteromers. The second case concerns the identification of PPI inhibitors (14).

Taken together, this Research Topic provides an illustrative overview of the principles and applications of the BRET technology that should be of interest for any scientist interested in monitoring PPI in intact cells.
